# Intermittent short-duration reoxygenation relieves high-altitude pulmonary hypertension via NOX4/H_2_O_2_/PPAR-γ axis

**DOI:** 10.1042/CS20231508

**Published:** 2024-01-30

**Authors:** Shaohua Li, Qiang Lyu, Qixin Shi, Yungang Bai, Xinling Ren, Jin Ma

**Affiliations:** 1 Department of Aerospace Physiology, Air Force Medical University, Xi’an 710032, China; 2 Department of Nephrology, Chinese PLA General Hospital, State Key Laboratory of Kidney Diseases, National Clinical Research Center for Kidney Diseases, Beijing 100853, China; 3 Department of Respiratory and Critical Care Medicine, Shenzhen University General Hospital, Shenzhen 518071, China

**Keywords:** hypobaric hypoxia, intermittent short-duration reoxygenation, NADHP oxidase 4, pulmonary hypertension

## Abstract

High-altitude pulmonary hypertension (HAPH) is a severe and progressive disease that can lead to right heart failure. Intermittent short-duration reoxygenation at high altitude is effective in alleviating HAPH; however, the underlying mechanisms are unclear. In the present study, a simulated 5,000-m hypoxia rat model and hypoxic cultured pulmonary artery smooth muscle cells (PASMCs) were used to evaluate the effect and mechanisms of intermittent short-duration reoxygenation. The results showed that intermittent 3-h/per day reoxygenation (I3) effectively attenuated chronic hypoxia-induced pulmonary hypertension and reduced the content of H_2_O_2_ and the expression of NADPH oxidase 4 (NOX4) in lung tissues. In combination with I3, while the NOX inhibitor apocynin did not further alleviate HAPH, the mitochondrial antioxidant MitoQ did. Furthermore, in PASMCs, I3 attenuated hypoxia-induced PASMCs proliferation and reversed the activated HIF-1α/NOX4/PPAR-γ axis under hypoxia. Targeting this axis offset the protective effect of I3 on hypoxia-induced PASMCs proliferation. The present study is novel in revealing a new mechanism for preventing HAPH and provides insights into the optimization of intermittent short-duration reoxygenation.

## Introduction

High-altitude pulmonary hypertension (HAPH) is a severe and progressive disease caused by chronic hypoxia and subsequent pulmonary vascular remodeling. No totally effective cure is currently available owing to an incomplete understanding of hypoxic vascular remodeling [1]. It is believed that hypoxia-induced diseases can be prevented by treating hypoxia directly. Studies have shown that intermittent short-duration reoxygenation can effectively alleviate pulmonary hypertension and right ventricular hypertrophy (RVH) induced by chronic hypoxia [2], and simulating oxygen supply by descending altitude achieved similar results as oxygen supply at high altitude in rats [3]. Furthermore, oxygen supply is currently an available countermeasure to high-altitude hypoxia. However, the underlying mechanisms of intermittent short-duration reoxygenation are unclear, and exploring the mechanism will provide insights into improving the oxygen supply protocol.

Our previous study has shown that the efficacy of intermittent short-duration reoxygenation is closely related to the level of oxidative stress in lung tissues. Specifically, increasing the frequency of reoxygenation from three times per day to six times per day aggravated HAPH instead due to the increased oxidative stress in the lung tissues [2]. Hence, reactive oxygen species (ROS) might play important roles in the effect of intermittent short-duration reoxygenation. There are two main sources of ROS production: the electron transport chain (ETC) in mitochondria and NADPH oxidase (NOX) in the cytoplasm [4]. Studies have shown that NOX4 is the most widely expressed NOX in the cardiovascular system [5]. Our pre-experiment showed that I3 reduced the expression of NOX4 and the level of H_2_O_2_ in lung tissues. NOX4 is an important source of H_2_O_2_. Hence, it is suggested that NOX4-mediated changes in H_2_O_2_ are involved in the role of I3.

NOX4 has been indicated to be involved in the process of hypoxic pulmonary hypertension, and the elevation of its expression is associated with pulmonary vascular remodeling [6]. Under chronic hypoxia, the expression of NOX4 in lung tissues and pulmonary arteries of mice is increased, as NOX4 is one of the target genes regulated by hypoxia inducible factor 1α (HIF-1α) [7]. Furthermore, the specific NOX4 inhibitor GKT137831 can attenuate chronic hypoxic pulmonary hypertension in mice [8]. The characteristic of NOX4 is directly and constitutively producing H_2_O_2_ without superoxide dismutase (SOD) and without activation [9]. Hence, the reduction in NOX4 expression and H_2_O_2_ content might account for the protective effect of I3 in relieving HAPH.

Studies have shown that the role of NOX4 in hypoxic pulmonary hypertension is associated with peroxisome proliferator-activated receptor γ (PPAR-γ) [10]. PPAR-γ is an important regulator of lipid and glucose metabolism and modulates the growth and proliferation of smooth muscle cells. The reduction in the expression of PPAR-γ participates in the pathogenesis of pulmonary hypertension by promoting the proliferation of PASMCs [11]. In addition, administration of the PPAR-γ agonist rosiglitazone attenuates pulmonary hypertension, right ventricular hypertrophy, and pulmonary vascular remodeling in chronic hypoxia-exposed rats [12]. PPAR-γ can be down-regulated by H_2_O_2_, which can be directly produced by NOX4 [10].

In summary, we assume that the NOX4/H_2_O_2_/PPAR-γ axis might be involved in the protective effect of I3. To verify the hypothesis above, we used animal hypobaric chambers to simulate hypobaric hypoxia at an altitude of 5,000 m and reduced the pressure to ground level (∼400 m) three times per day for one hour each time to simulate the oxygen supply at high altitude. In addition, we used a triple gas incubator to establish a cellular hypoxia model. The role of NOX4 and PPAR-γ in the protective effect of I3 was initially examined.

## Methods

### Rat model

**Figure 1:**
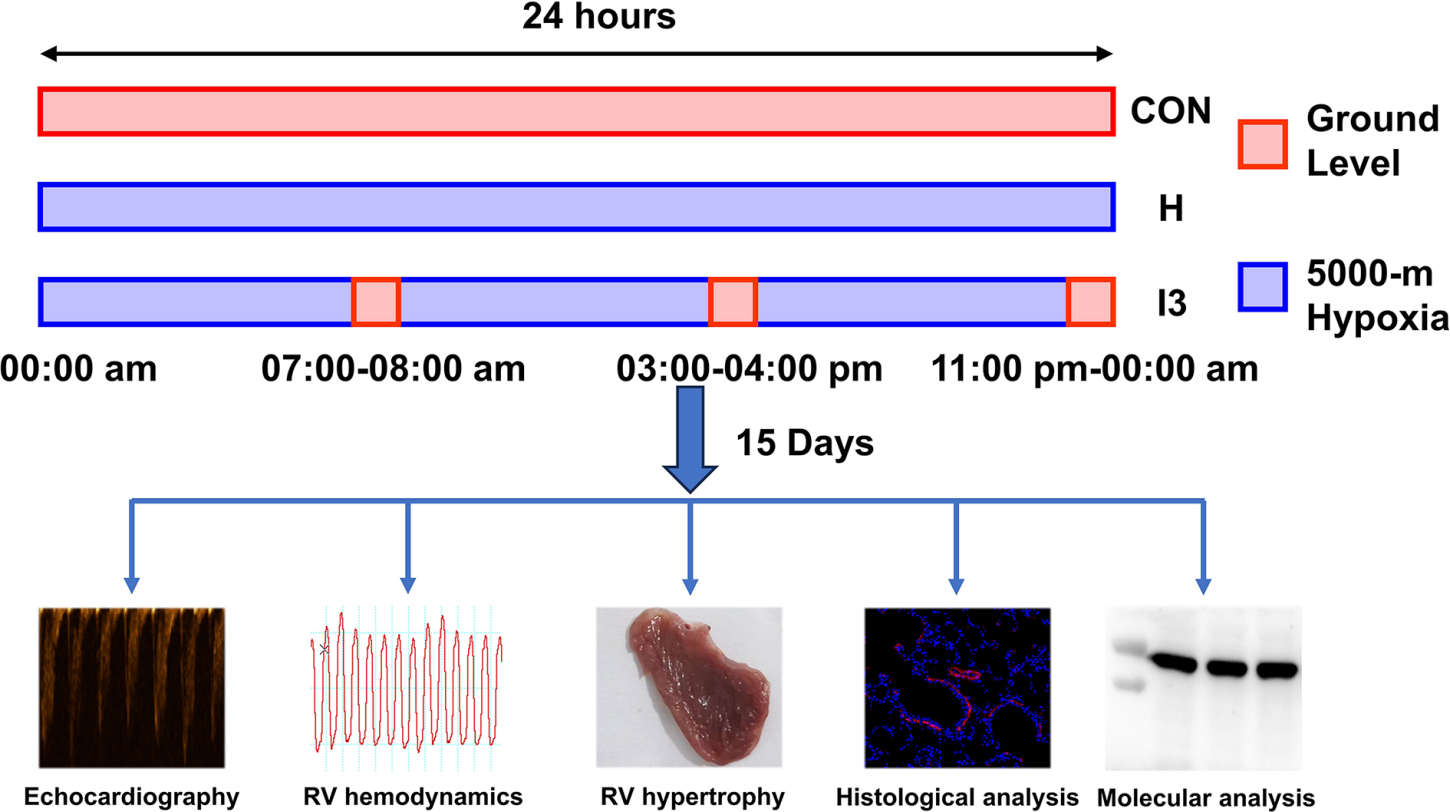
Protocol of animal experiments

Sprague‒Dawley (SD) rats (approximately 7 to 8 weeks old) were purchased from the Animal Center of the Air Force Medical University. After several days of adaptive breeding, the rats were transferred to animal hypobaric chambers to simulate an altitude of 5000 meters above sea level (approximately 405 mmHg) for 15 days. Excess CO_2_ and water vapor in the chambers were separately absorbed by sodium lime and silica gel powder. The chambers were descended to replenish food and water and to clean the cage every 3 days. For the I3 group, the chambers were descended to ground level (Xi’an China, approximately 400 m) for 1 h and three times per day with an interval of 7 h to simulate the oxygen supply on the plateau, and the times for reoxygenation were 7:00–8:00, 15:00–16:00, and 23:00–0:00 (Figure 1). This protocol of reoxygenation was adopted because I3 can achieve better protective effect on HAPH than I2 and I6 according to our previous study [2].

To inhibit different sources of ROS in rats attached to I3, MitoQ (MCE, HY-100116A) and apocynin (MCE, HY-N0088) were administered by gavage at doses of 10 mg/kg/d (MitoQ was dissolved in 0.9% NaCl solution to 3 mg/ml) and 200 mg/kg/d (Apocynin was suspended in 0.9% NaCl solution to 60 mg/ml), respectively. The rats in the control group were normally fed at ground level. All rats were bred under light–dark cycles of 12 h.

### Hemodynamic measurement and tissue preparation

Ultrasonic measurement was conducted before hypoxia and every three days during hypoxia by VINNO 6 LAB. The measurements included pulmonary artery flow Doppler and B-mode ultrasound of the right ventricle. During ultrasonic measurements, the rats were anesthetized by isoflurane inhalation (3% for induction and 1% for maintenance). The long axis section of the left sternum edge was used to measure the ratio of pulmonary artery blood flow acceleration time to pulmonary artery blood ejection time (PAT/PET), and the short axis section of the sternum was measured to obtain the end diastolic thickness of the anterior wall of the right ventricle (RVAWd) [13].

After 15 days of hypoxia and final ultrasonic measurement, the right jugular vein was surgically exposed, and a PE50 plastic catheter connected to a pressure sensor and filled with heparin sodium solution was inserted into the jugular vein and slowly inserted into the right ventricle to record the right ventricular systolic pressure (RVSP). After the measurement was completed, the rats were sacrificed by blood collection from the abdominal aorta and perfused with saline to flush the residual blood from the lungs. Heart and lung tissues were collected for subsequent experiments. Then, the right ventricle was removed and weighed separately. The ratio of the right ventricle weight to the left ventricle plus the ventricular septum weight is the right ventricular hypertrophy index (RVHI).

### Oxidative stress indicator assay

For dihydroethidium (DHE) staining, after flushing blood from the lung with cold 0.9% NaCl solution, fresh rat lung tissue was embedded in optimal cutting temperature compound to make frozen sections. Then, the sections were stained with DHE probe according to the instructions. After the staining was completed, a fluorescence microscope was used to obtain random field of view and ImageJ software was used to quantify the fluorescence intensity after converting to gray mode.

For other oxidative stress-related indices, different kits purchased from Nanjing Jiancheng Bioengineering Institute were used to detect malondialdehyde (MDA) content (A003-1-2), H_2_O_2_ content (A064-1-1), catalase (CAT) activity (A007-1-1), SOD activity (A001-2-2) and NADH/NAD^+^ (A114-1-1) in lung tissues and MDA content and H_2_O_2_ content in serum. Before the test, rat lung tissues were ground into homogenate with 0.9% NaCl solution for MDA, H_2_O_2_, CAT and SOD tests. For the NADH/NAD^+^ test, rat lung tissues were ground into homogenate by the lysis buffers contained in the kit. All tests in lung tissue homogenate and serum were performed according to the instructions provided by the manufacturer. In brief, mix the homogenate or serum and the reagents in the kit in the specified order and proportion, and some processes may require heating. Finally, a microplate reader was used to detect the OD value, which was used to calculate content or activity. 8-OHG in serum was tested by ELISA (Cloud-Clone, CEA660Ge).

### PASMCs culture and hypoxic cell model

Rat pulmonary artery smooth muscle cells and suitable growth medium were purchased from iCell Bioscience Inc., Shanghai. Before the modeling, PASMCs were seeded in 96-well plates (2000 cells per well, for CCK8 test) or 6-well plates (50000 cells per well, for WB test). After cell attachment for a night, the cell hypoxia model was established by a hypoxic incubator. PASMCs were cultured in 1% O_2_ for 56 h. For I3, the cells were transferred to an ordinary carbon dioxide incubator for reoxygenation. The transfer time and frequency were the same as those of the animal model. The 56-h reoxygenation model contains seven cycles of hypoxia-reoxygenation. At the end of culture, protein was extracted, or the OD value of the CCK8 test was read. The drugs used for PASMCs included 100 or 200 μM dimethyloxallyl glycine (DMOG, a stabilizer of HIF-1α, MCE, HY-15893), 3 mM (0.01%) H_2_O_2_ (Sigma, 323381), 5 μM GLX351322 (an inhibitor of NOX4, MCE, HY-100111), 20 μM T0070907 (an inhibitor of PPAR-γ, MCE, HY-13202), 5 μM Rosiglitazone (an agonist of PPAR-γ, MCE, HY-17386), and 0.1 μM MitoQ (a mitochondria-targeted antioxidant, MCE, HY-100116A). DMOG was dissolved in PBS, and the other drugs were dissolved in Dimethyl sulfoxide (DMSO). At the beginning of modeling, the culture medium was replaced with culture medium containing a certain concentration of drugs.

### Western blot assay

Western blotting was conducted as previously described. Briefly, lung tissues or cells were lysed by Tissue Protein Extraction Reagent (78510, Thermo) and RIPA Buffer (89900, Thermo) with Protease Inhibitor Cocktail (1:100, HY-K0010, MCE). The protein concentrations of lysates were detected using a BCA protein assay kit (23225, Thermo). After protein denaturation by heating, samples containing 20 μg protein were subsequently electrophoresed in 4–12% Bis-Tris PAGE gels and were then transferred to polyvinylidene difluoride (PVDF) membranes. Unspecific antibody binding was blocked by incubating the membranes for 3 h with 5% BSA in TBST at room temperature. Subsequently, the membranes were incubated overnight at 4°C with primary antibodies against the following proteins: NOX2 (1:1000, ab129068, Abcam, rabbit), NOX4 (1:1000, 14347-1-AP, Proteintech, rabbit), PPAR-γ (1:1000, BS6442, Bioworld, rabbit), HIF-1α (1:1000, ab1, Abcam, mouse), proliferating cell nuclear antigen (PCNA) (1:1000, 13110, CST, rabbit), and GAPDH (1:1000, D16H11, CST, rabbit). The membranes were then washed and incubated with goat anti-mouse secondary antibody (1:10000, SA-10010, Incellgene) or goat anti-rabbit secondary antibody (1:10000, SA-10011, Incellgene) for 90 min. After washing, bound antibodies were detected using enhanced chemiluminescence detection reagents (IC-5009-100, Incellgene) in a Gel Image Analyzing System (Tanon-4200; Tanon Science & Technology, Shanghai, China). Densitometry analysis of bands was conducted using ImageJ software.

### Immunofluorescence

The fixed lung tissues were embedded in paraffin, and cross sections (5 μm) were prepared. Then, the lung sections were used for immunostaining. In brief, after dewaxing, antigen retrieval (pH 6.0 citrate buffer and heating) and blocking by goat serum, the lung sections were stained with antibody against α-smooth muscle actin (α-SMA) (1:500, ab7817, Abcam, Mouse) and CD31 (1:500, GB11063-2, Servicebio, Rabbit) overnight. Subsequently, Goat anti-Mouse IgG (1:1000, SAG59401-100 antiProtech), Goat anti-Rabbit IgG (1:1000, SAG48802, antiProtech) and DAPI were incubated sequentially. After staining, observation and photograph was taken by a fluorescence microscope (EVOS M5000, ThermoFisher). Analysis was performed on vessels with an external diameter of 25–75 μm. Specifically, a vessel was considered partially or fully muscularized if it exhibited α-SMA**^+^** throughout at least half or the complete circumference of the vessel cross-section, respectively. Pulmonary vascular wall thickness was detected by measuring the medial thickness of distal pulmonary vessels with a diameter of 100–200 μm from lung sections stained with α-SMA. The percent wall thickness was expressed as the ratio of medial wall area to the vessel area [14].

To detect the proliferation level of PASMCs, α-SMA and Ki67 co-staining was conducted. The primary antibodies were α-SMA (1:500, ab7817, Abcam, Mouse) and Ki67 (1:500, GB11063-2, Servicebio, Rabbit). The ratios of Ki67-positive arteries were detected in vessels with an external diameter of 25–200 μm from lung sections co-immunostained with α-SMA and Ki67.

### Statistical analysis

Data are expressed as mean ± SEM. Statistical analysis was performed using GraphPad Prism 9.5. Echocardiogram measurements were analyzed by two-way ANOVA with post hoc Tukey’s HSD analysis. In the other experiments, data were compared using *t-*tests for two groups or one-way ANOVA with post hoc Tukey’s HSD analysis for three groups. *P-*value <0.05 was considered to indicate statistical significance.

## Results

### I3 alleviated HAPH and RVH

**Figure 2:**
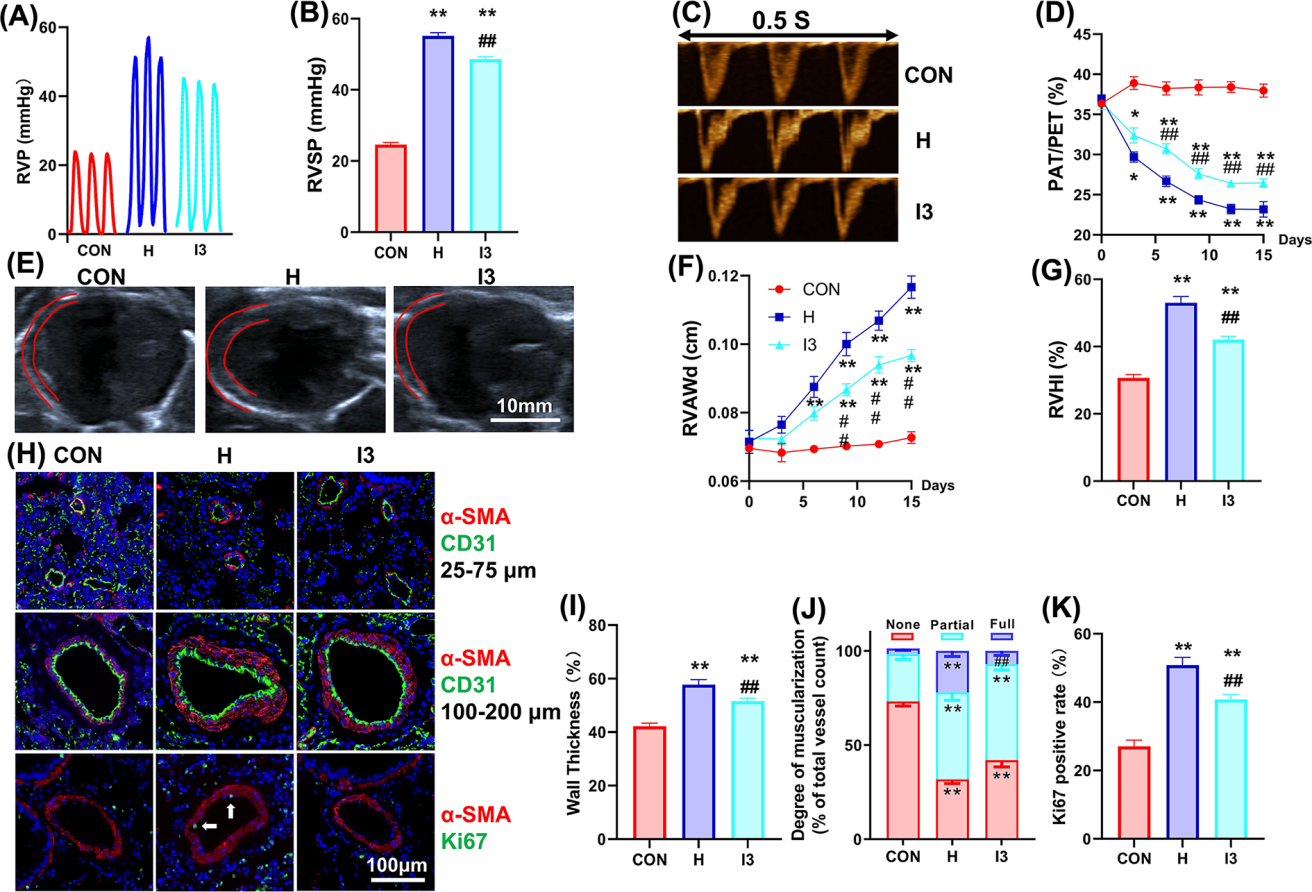
I3 alleviated HAPH and right ventricular hypertrophy (**A**) Representative traces of right ventricular pressure. (**B**) Right ventricular systolic pressure values in different groups. (**C**) Representative Doppler image of pulmonary blood flow. (**D**) Variation curve of the ratio of pulmonary artery acceleration time to pulmonary artery ejection time. (**E**) Representative ultrasound image of heart section; (**F**) Variation curve of right ventricle end-diastolic anterior wall thickness. (**G**) Right ventricular hypotrophy index values in different groups. (**H**) Representative image of immune fluorescence staining for α-SMA, CD31, and Ki67 of lung tissue sections. (**I**) Wall thickness of pulmonary vessels with a diameter of 100-200 μm from lung sections immune stained with α-SMA. Percent wall thickness is expressed as the medial wall area divided by the area of the vessel. (**J**) Percentage of muscularization of distal pulmonary arteries with a diameter of 25–75 μm. (**K**) Ki67 positive rate in distal pulmonary arteries. The results are expressed as the mean ± SEM, *n* = 8 in each group. *P<0.05, **P<0.01 vs. CON; ^#^*P*<0.05, ^##^*P*<0.01 vs. H. CON for the control group, H for the chronic hypoxia group, I3 for the intermittent short-duration reoxygenation group.

To verify the protective effect of restoring atmospheric pressure in the simulated oxygen supply model, we examined the degree of HAPH in the different groups. I3 partially alleviated the chronic hypoxia-induced changes in the hemodynamics of the right heart. Specifically, chronic hypoxia significantly elevated the right ventricular systolic pressure (RVSP), and I3 partially reversed this effect (Figure 2A,B). Doppler ultrasound showed that the ratio of pulmonary acceleration time to pulmonary ejection time (PAT/PET) gradually decreased during chronic hypoxia, and I3 slowed the rate of this decrease and resulted in an elevated final value (Figure 2C,D). B-mode ultrasound showed that chronic hypoxia gradually thickened the anterior wall of the right ventricle, and I3 slowed the rate of thickening and decreased the ultimate thickness (Figure 2E,F). In addition, chronic hypoxia significantly increased the RVHI, and I3 partially reversed this change (Figure 2G). Regarding the muscularization of distal pulmonary arterioles, I3 reduced the thickness of arterioles with a diameter between 100 and 200 μm, and the total muscularization rate of arterioles with a diameter between 25 and 75 μm was elevated by chronic hypoxia (Figure 2H-J). The detection of Ki67 *in vivo* indicated that chronic hypoxia promoted the proliferation of PASMCs and I3 partially restored this effect (Figure 2H,K).

### The reduced H_2_O_2_ content in lung tissues participated in the protecting role of I3

**Figure 3:**
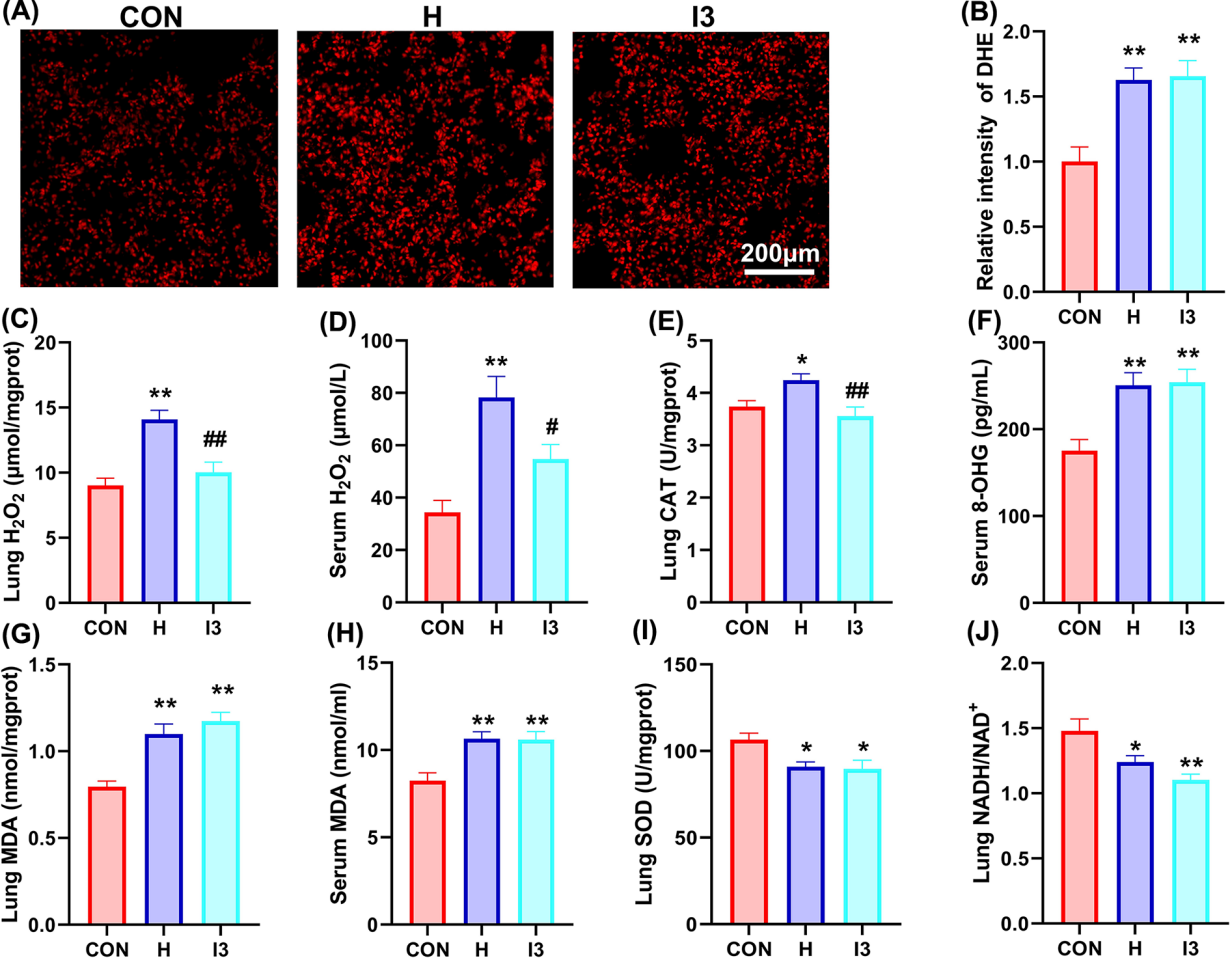
The reduced H_2_O_2_ content in lung tissues participated in the protecting role of I3 (**A**) Representative image of dihydroethidium staining in lung tissue sections. (**B**) Statistical analysis of the fluorescence intensity of dihydroethidium. (**C**) The content of H_2_O_2_ in lung tissues. (**D**) The content of H_2_O_2_ in serum. (**E**) The activity of catalase in lung tissues. (**F**) The content of 8-hydroxyguanosine in serum. (**G**) The content of malondialdehyde in lung tissues. (**H**) The content of malondialdehyde in serum. (**I**) The activity of superoxide dismutase in lung tissues. (**J**) The ratio of NADH/NAD^+^ in lung tissues. The results are expressed as the mean ± SEM, *n*=6 in each group for dihydroethidium staining, *n*=8 for other oxidative indices. *P<0.05, **P<0.01 vs. CON; ^#^*P*<0.05, ^##^*P*<0.01 vs. H.

To explore the mechanism by which I3 ameliorates HAPH, we examined the oxidative stress of the different groups. DHE staining of lung sections showed that chronic hypoxia increased the fluorescence intensity of O^2−^, but I3 did not reduce the fluorescence intensity of O^2−^ (Figure 3A,B). In addition, chronic hypoxia increased the contents of H_2_O_2_ and MDA in both lung tissues and serum, increased the activity of CAT in lung tissues and the content of 8-OHG in serum. Besides, chronic hypoxia reduced the activity of SOD and the ratio of NADH/NAD^+^ in lung tissues. I3 reduced the content of H_2_O_2_ and the activity of CAT in lung tissues and decreased the content of H_2_O_2_ in serum (Figure 3C–E), while I3 did not obviously restore other indices affected by chronic hypoxia (Figure 3F–J).

### The restored expression of NOX4 by I3 explained the reduced H_2_O_2_ content and influenced the expression of PPAR-γ

**Figure 4:**
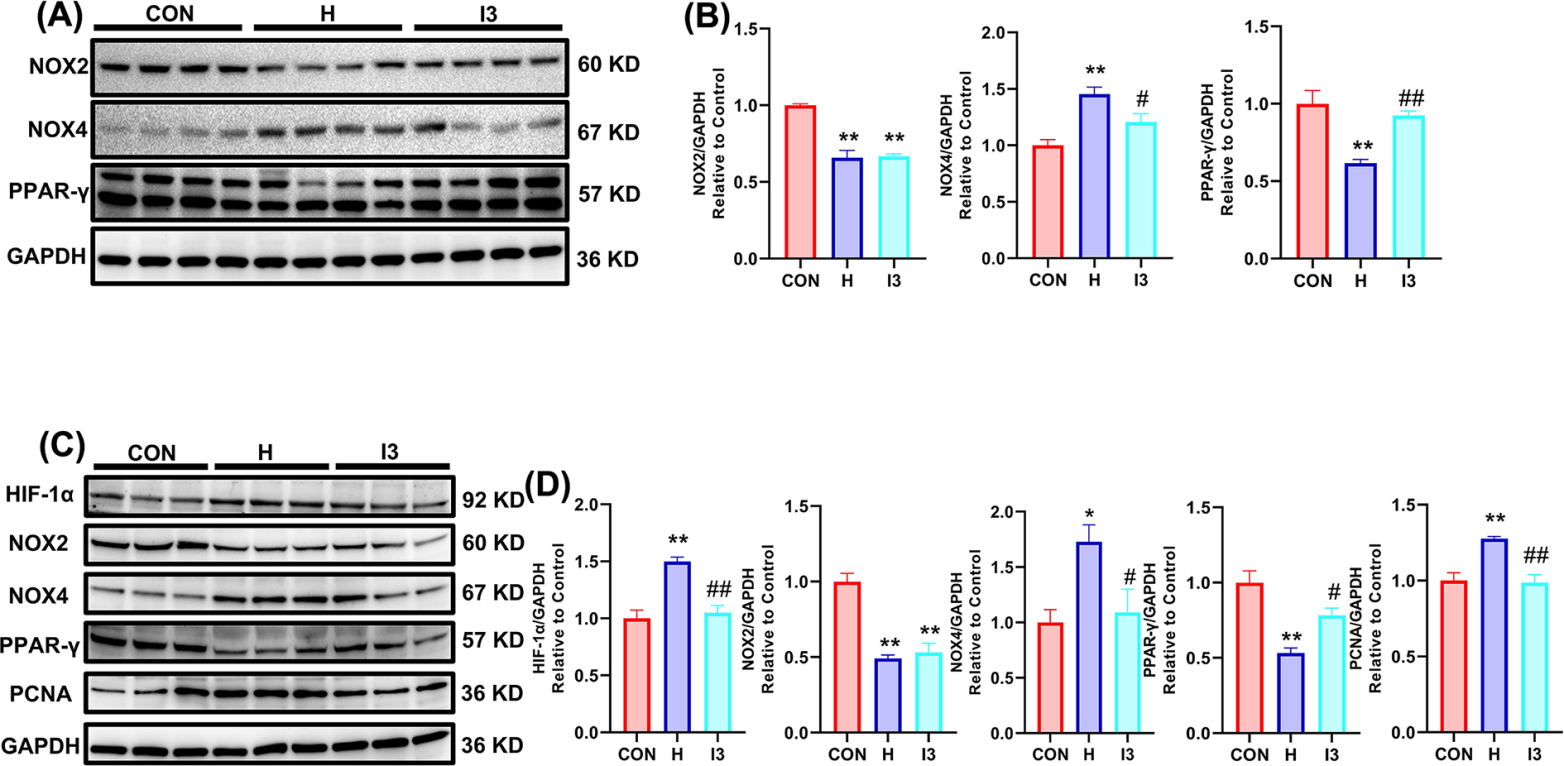
The restored expression of NOX4 by I3 explained the reduced H_2_O_2_ content and influenced the expression of PPAR-γ (**A**) Representative Western blotting bands for NOX2, NOX4 and PPAR-γ in lung tissues. (**B**) Statistical analysis of NOX2, NOX4, and PPAR-γ expression in lung tissues. (**C**) Western blotting bands for HIF-1α, NOX2, NOX4, PPAR-γ, and PCNA in cultured PASMCs. (**D**) Statistical analysis of HIF-1α, NOX2, NOX4, PPAR-γ, and PCNA expression in cultured PASMCs. The results are expressed as the mean ± SEM, *n*=4 in each group for lung tissues, *n*=3 for PASMCs. *P<0.05, **P<0.01 vs. CON; ^#^*P*<0.05, ^##^*P*<0.01 vs. H.

To further explore the source of H_2_O_2_, we observed changes in the expression of H_2_O_2_-related proteins in lung tissues. Western blotting assays with lung tissue homogenates showed that chronic hypoxia up-regulated the expression of NOX4 and downregulated the expression of PPAR-γ and NOX2. I3 partially reversed the changes in NOX4 and PPAR-γ expression but had no effect on NOX2 expression (Figure 4A,B).

To verify these changes in PASMCs, which are one of the main cell types affected by HAPH, we observed changes in the expression of these proteins in cultured PASMCs. The results showed that exposure to 1% O_2_ for 56 h increased the expression of HIF-1α, NOX4, and PCNA and decreased the expression of NOX2 and PPAR-γ. I3 partially reversed the changes in HIF-1α, NOX4, PPAR-γ, and PCNA expression that had been affected by chronic hypoxia but did not restore NOX2 expression (Figure 4C,D).

### I3 alleviated hypoxia-induced proliferation of PASMCs via the HIF-1α/NOX4/H_2_O_2_/PPAR-γ axis

**Figure 5:**
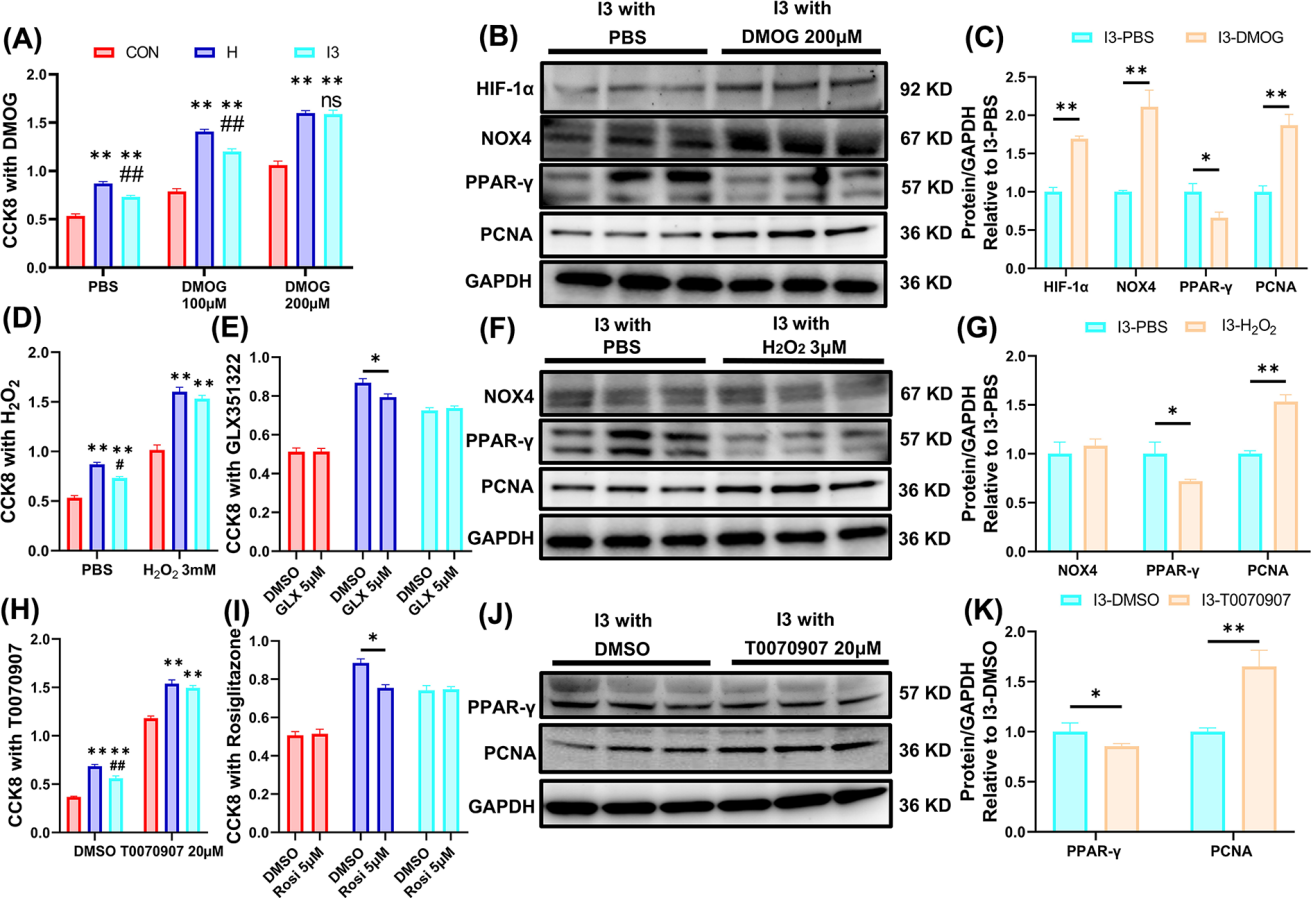
I3 alleviated hypoxia-induced proliferation of PASMCs via the HIF-1α/NOX4/H_2_O_2_/PPAR-γ axis (**A**) CCK-8 test of PASMCs incubated with DMOG. (**B**) Western blotting bands for HIF-1α, NOX4, PPAR-γ, and PCNA in PASMCs incubated with DMOG. (**C**) Statistical analysis of HIF-1α, NOX4, PPAR-γ, and PCNA in PASMCs incubated with DMOG. (**D**) CCK-8 test of PASMCs incubated with H_2_O_2_. (**E**) CCK-8 test of PASMCs incubated with the NOX4 inhibitor GLX351322. (**F**) Western blotting bands for NOX4, PPAR-γ, and PCNA in PASMCs incubated with H_2_O_2_. (**G**) Statistical analysis of NOX4, PPAR-γ, and PCNA in PASMCs incubated with H_2_O_2_. (**H**) CCK-8 test of PASMCs incubated with the PPAR-γ inhibitor T0070907. (**I**) CCK-8 test of PASMCs incubated with the PPAR-γ agonist rosiglitazone. (**J**) Western blotting bands for NOX4, PPAR-γ, and PCNA in PASMCs incubated with T0070907. (**K**) Statistical analysis of PPAR-γ and PCNA in PASMCs incubated with T0070907. The results are expressed as the mean ± SEM, *n*=10 in each group for the CCK-8 test, *n*=3 for the Western blotting assay. *P<0.05, **P<0.01 vs. CON; ^#^*P*<0.05, ^##^*P*<0.01 vs. H.

After exposure to 1% O_2_ hypoxia for 56 h, the number of PASMCs increased, and I3 partially reversed this change. Incubation with 100 μM DMOG increased the number of PASMCs in all three groups. When the DMOG concentration was increased to 200 μM, the difference between the H and I3 groups disappeared (Figure 5A). Western blotting assays showed that I3 plus 200 μM DMOG altered the expression of HIF-1α, NOX4, PPAR-γ and PCNA in a manner that was similar to continuous hypoxia (Figure 5B,C).

Incubation with 3 mM H_2_O_2_ increased the PASMC numbers in all three groups, eliminating the difference between the H and I3 groups (Figure 5D). Incubation with the specific NOX4 inhibitor GLX351322 reduced the cell count only in the H group (Figure 5E). Western blotting assays showed that I3 plus 3 mM H_2_O_2_ down-regulated PPAR-γ and up-regulated PCNA but had no obvious effect on NOX4 (Figure 5F,G).

Incubation with 20 μM T0070907, which is a specific PPAR-γ inhibitor, increased the numbers of PASMCs in all three groups, eliminating the difference between the H and I3 groups (Figure 5H). In addition, incubation with 5 μM rosiglitazone, which is a PPAR-γ agonist, reduced the cell count only in the H group (Figure 5I). Western blotting assays showed that treatment with I3 and 20 μM T0070907 down-regulated PPAR-γ and up-regulated PCNA (Figure 5J,K).

### I3 in combination with NOX4 inhibition did not further alleviate HAPH in rats

**Figure 6:**
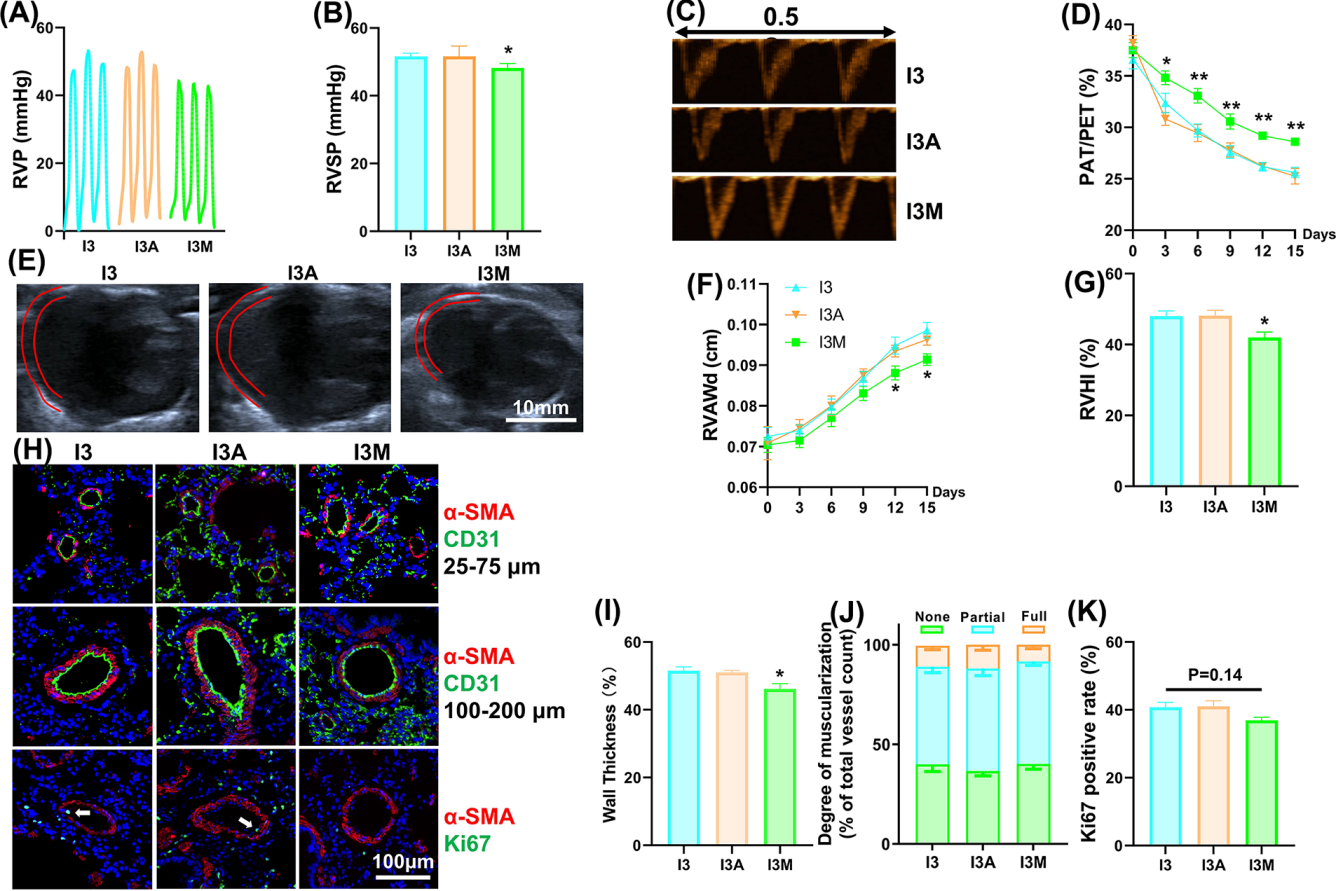
I3 in combination with NOX4 inhibition did not further alleviate HAPH in rats (**A**) Representative traces of right ventricular pressure. (**B**) Right ventricular systolic pressure values in different groups. (**C**) Representative Doppler image of pulmonary blood flow. (**D**) Variation curve of the ratio of pulmonary artery acceleration time to pulmonary artery ejection time. (**E**) Representative ultrasound image of heart section. (**F**) Variation curve of right ventricle end-diastolic anterior wall thickness. (**G**) Right ventricular hypotrophy index values in different groups. (**H**) Representative image of immune fluorescence staining for α-SMA, CD31 and Ki67 of lung tissue sections. (**I**) Wall thickness of pulmonary vessels with a diameter of 100–200 μm from lung sections immune stained with α-SMA. Percent wall thickness is expressed as the medial wall area divided by the area of the vessel. (**J**) Percentage of muscularization of distal pulmonary arteries with a diameter of 25–75 μm. (**K**) Ki67 positive rate in distal pulmonary arteries. The results are expressed as the mean ± SEM, *n* = 8 in each group. The results are expressed as the mean ± SEM, *n* = 8 in each group. *P<0.05, **P<0.01 vs. I3. I3A for I3 with apocynin 200 mg/kg/d, I3M for I3 with MitoQ 10 mg/kg/d.

To further validate the role of NOX4 in I3, we treated rats that were subjected to I3 with two kinds of antioxidants. One antioxidant was the NOX inhibitor apocynin, and the other was the mitochondrial antioxidant MitoQ. The results showed that MitoQ-treated rats exhibited further amelioration of HAPH, and apocynin-treated rats showed no significant difference from vehicle-treated rats. Specifically, RVSP was further reduced by MitoQ but not apocynin (Figure 6A,B). Doppler ultrasound showed that MitoQ slowed the decrease in PAT/PET and resulted in an increase in the final value, while apocynin had no obvious effect on PAT/PET (Figure 6C,D). B-mode ultrasound showed that MitoQ reduced the thickness of the anterior wall of the right ventricle, but apocynin did not (Figure 6E,F). In addition, MitoQ, but not apocynin, further reduced the RVHI (Figure 6G). Regarding the muscularization of distal arterioles, MitoQ further reduced the wall thickness of vessels with a diameter of 100–200 μm but had no significant effect on the degree of muscularization of vessels with a diameter of 25–75 μm (Figure 6H–J). Besides, MitoQ has a tendency to further reduce the proliferation of PASMCs (Figure 6H,K).

### Reduced mitochondrial H_2_O_2_ content explains the MitoQ-mediated alleviation of HAPH when combined with I3

**Figure 7:**
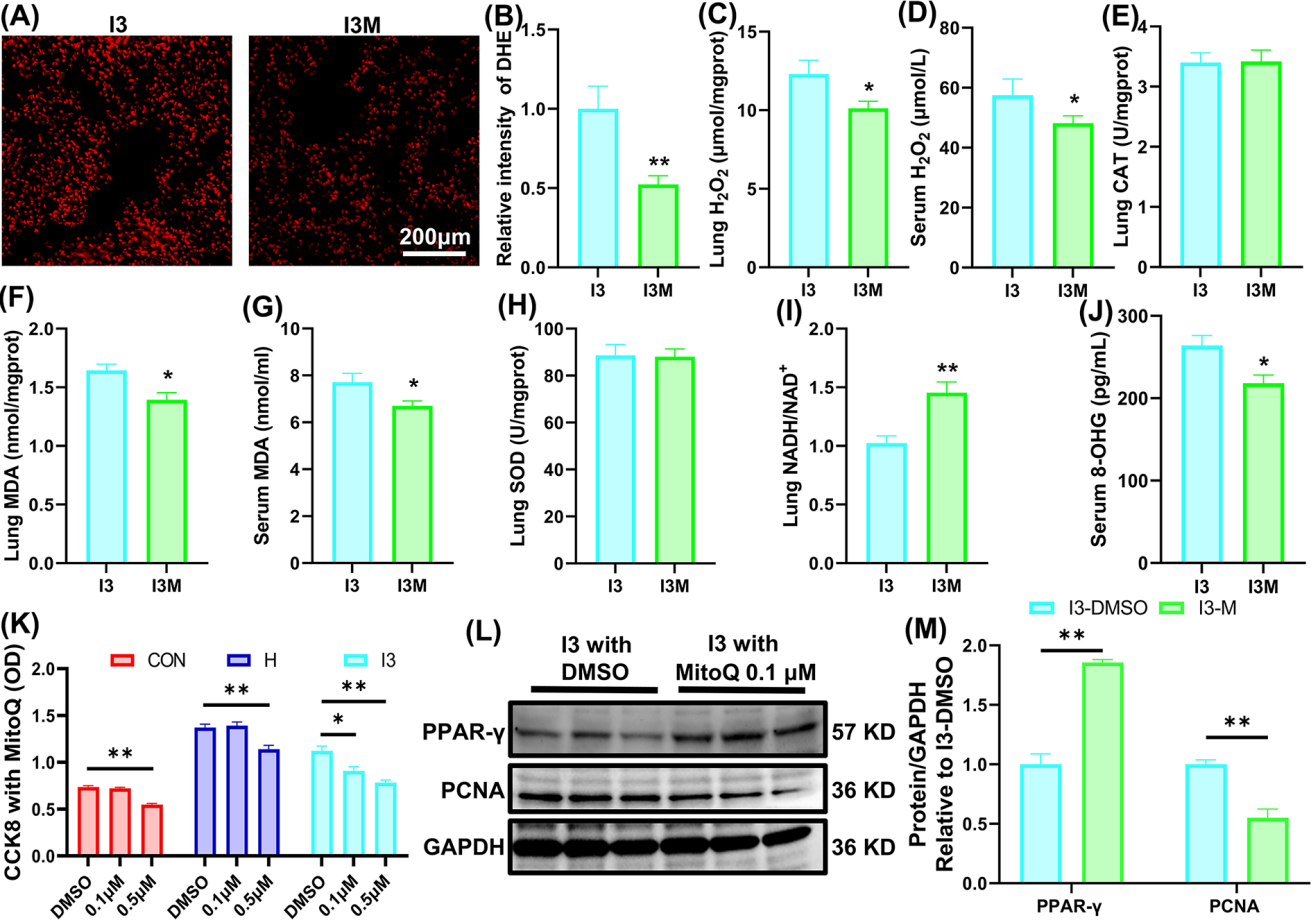
Reduced mitochondrial H_2_O_2_ content explains the MitoQ-mediated alleviation of HAPH when combined with I3 (**A**) Representative image of dihydroethidium staining in lung tissue sections. (**B**) Statistical analysis of the fluorescence intensity of dihydroethidium. (**C**) The content of H_2_O_2_ in lung tissues. (**D**) The content of H_2_O_2_ in serum. (**E**) The activity of catalase in lung tissues. (**F**) The content of malondialdehyde in lung tissues. (**G**) The content of malondialdehyde in serum. (**H**) The activity of superoxide dismutase in lung tissues. (**I**) The ratio of NADH/NAD^+^ in lung tissues. (**J**) The content of 8-hydroxyguanosine in serum. (**K**) CCK-8 test of PASMCs incubated with MitoQ. (**L**) Western blotting bands for PCNA and PPAR-γ in cultured PASMCs incubated with MitoQ. (**M**) Statistical analysis of PCNA and PPAR-γ in PASMCs incubated with MitoQ. The results are expressed as the mean ± SEM, *n*=6 in each group for dihydroethidium staining, *n*=8 for other oxidative indices, *n*=10 for CCK8 test, *n*=3 for Western blotting assay. *P<0.05, **P<0.01 vs. I3.

To explore whether MitoQ further alleviated HAPH by reducing mitochondrial H_2_O_2_ content, we examined the oxidative state after MitoQ administration. The DHE staining results showed that MitoQ decreased the fluorescence intensity of DHE (Figure 7A,B). Oxidative index kits showed that MitoQ further reduced the content of H_2_O_2_ in lung tissues and serum but had no influence on the activity of CAT and SOD in lung tissues (Figure 7C–E,H). In addition, MitoQ reduced the content of MDA in both lung tissues and serum and decreased the content of 8-OHG in serum (Figure 7F,G,J). Finally, MitoQ increased the NADH/NAD^+^ ratio (Figure 7I).

In cultured PASMCs, 0.1 μM MitoQ reduced the cell count of the I3 group but did not reduce the cell count of the CON and H groups. Increasing the concentration of MitoQ to 0.5 μM nonspecifically reduced the cell count of all three groups (Figure 7K). In addition, Western blotting assays showed that 0.1 μM MitoQ further decreased the expression of PCNA and increased the expression of PPAR-γ in PASMCs that were subjected to I3 (Figure 7L,M).

## Discussion

Previous animal experiments have shown that intermittent 3-h per day reoxygenation is a promising and practical measure to combat HAPH and the current study also reached similar results. We chose this protocol of reoxygenation because we have tried four protocols of reoxygenation including I2, I3, I6, C3 (continuous 3-h per day) and I3 reached the best protective effect. Although I3 could alleviate HAPH and RVH, the underlying mechanism was not clear. Understanding the underlying mechanism can help us improve the protocol and ensure the safety of reoxygenation. The goals of the current study were to further define the role of NOX4 and PPAR-γ in the protective effects of I3. The primary conclusion from our results is that the NOX4/H_2_O_2_/PPAR-γ axis participates in the protective effect of I3 on HAPH.

Oxidative stress is a critical pathophysiological process in hypoxia and has been extensively studied [15]. However, there is still a major debate about the changes in ROS under hypoxic conditions [16]. One hypothesis is that hypoxia inhibits oxidative phosphorylation and reduces ROS production and another is that hypoxia increases ROS by altering the structure of mitochondrial ETC [17]. The reason for this debate may be the complexity of the types, sources, and subcellular localization of ROS [18]. The current study found that chronic hypoxia increased the content of oxidative metabolites, but I3 did not reduce the content of all kinds of metabolites, and only the content of H_2_O_2_ was restored. This result reminds us that the level of oxidative stress is elevated during hypoxia, but the specific source and localization are not clear. The restored content of H_2_O_2_ suggested that it can be involved in the protective roles of I3.

The role of NOX4 and PPAR-γ in hypoxic pulmonary hypertension has been well proven. The elevated expression of NOX4 by hypoxia participates in the proliferation of pulmonary artery smooth muscle cells and pulmonary artery endothelial cells [19]. However, another study has shown that *nox4* knockout mice did not develop milder hypoxic pulmonary hypertension [20]. We believe that NOX4 contributes to hypoxic pulmonary hypertension, but it can be compensated by other pathways. In our research, the elevated expression of NOX4 in lung tissues and cultured PASMCs was restored by I3. NOX4 is a NOX subtype that is consistently active without activation and can produce H_2_O_2_ directly without SOD. Hence, the restored content of H_2_O_2_ by I3 can be explained by the restored expression of NOX4. In addition, the restored content of H_2_O_2_ accounted for the expression of PPAR-γ, whose downregulation is vital in the process of pulmonary hypertension [21]. In cultured PASMCs, the HIF-1α stabilizer, exogenous H_2_O_2_ and PPAR-γ inhibitor all offset the protective effect of I3 on the proliferation of PASMCs.

A study has shown that the NOX inhibitor apocynin can alleviate hypoxic pulmonary hypertension in mice [22]. In the present study, the use of apocynin did not further alleviate HAPH in I3 group. This may be because I3 has already reduced the expression of NOX4, and the use of inhibitors cannot achieve better results when the expression is at a low level. MitoQ can further alleviate HAPH in rats attached to I3, suggesting that I3 may increase the level of mitochondrial ROS, which can also explain the effect of 6-time reoxygenation is worse than three-time reoxygenation in our previous study. Studies have shown that the mitochondrial ETC undergoes conformational changes and produces ROS during reoxygenation, which is one of the pathophysiological mechanisms of ischemia‒reperfusion injury [23]. The therapeutic effect of MitoQ on hypoxic pulmonary hypertension has been studied, and they found that MitoQ can alleviate RVH but not pulmonary vascular remodeling and pulmonary hypertension because they also found that 4 weeks of hypoxia decreased the content of mitochondrial superoxide in lung tissues [24]. The present study suggests that the mitochondrial ETC produces lower levels of O^2−^ during chronic hypoxia. However, under reoxygenation, the level of mitochondrial O^2−^ may increase, and a portion of O^2−^ will be converted into H_2_O_2_ by SOD2 and penetrate into the cytoplasm. MitoQ can reduce this part of H_2_O_2_ from non-NOX4 sources, which might account for MitoQ further relieving HAPH in rats attached to I3.

Based on our results, the changes in ETC derived ROS and NOX derived ROS are not the same on the condition of I3. Reducing mitochondria derived ROS is a method to enhance the protective effect of I3. Sleeping in oxygen enriched rooms on high altitude area has been proven to improve sleep quality and work ability [25]. But it has not yet been proven that this method has a protective effect on HAPH. According to our research, whether combining day-time intermittent reoxygenation with night-time reoxygenation can alleviate HAPH because low metabolic level at night may produce fewer mitochondrial ROS [26], which requires further research to clarify.

The limitations of the present study mainly lie in two aspects. (1) The present study only explored the NOX4/H_2_O_2_/PPAR-γ axis in PASMCs, while pulmonary artery endothelial cells and adventitial fibroblasts also play an important role in the development of pulmonary hypertension [27]. However, this mechanism in PASMCs can explain how I3 alleviates HAPH, and we will explore this mechanism in PAECs in our future research. (2) The mechanism of how MitoQ further alleviate HAPH in rats attached to I3 has not been researched deeply because this is not closely related to the main content of this study, and we will study it in the future.

In conclusion, we found that daily intermittent short-duration reoxygenation partially prevented pulmonary hypertension through regulating the NOX4/H_2_O_2_/PPAR-γ axis in PASMCs. The impacts of intermittent short-duration reoxygenation on ROS from NOX4 and mitochondria were different and mitochondria-targeted antioxidant can further enhance the protective effect of intermittent short-duration reoxygenation. This study is novel in revealing a new mechanism in preventing HAPH and giving insights into the optimization of intermittent short-duration reoxygenation.

## Clinical perspectives

- Intermittent short-duration reoxygenation is a promising countermeasure for hypoxic pulmonary hypertension and right ventricular hypertrophy, but the underlying mechanism is not clear and the protective effect should be further enhanced.
- We found that intermittent short-duration reoxygenation relieved pulmonary hypertension via NOX4/H2O2/PPAR-γ axis and further reducing non-NOX4 dereived H_2_O_2_ could further alleviated hypoxic pulmonary hypertension.
- Intermittent oxygen supply plus mitochondria targeted antioxidants could further alleviate hypoxic pulmonary hypertension.

## Data Availability

All relevant data of this study are available from the corresponding author upon reasonable request.

## Abbreviations

CAT: catalase
DHE: dihydroethidium
HAPH: high-altitude pulmonary hypertension
HIF-1α: hypoxia inducible factor 1α
NOX4: NADPH oxidase 4
PASMC: pulmonary artery smooth muscle cell
PPAR-γ: peroxisome proliferator-activated receptor γ
RVH: right ventricular hypertrophy
RVHI: right ventricular hypertrophy index
ROS: reactive oxygen species
ETC: electron transport chain
SOD: superoxide dismutase
RVAWd: end diastolic thickness of the anterior wall of the right ventricle
RVSP: right ventricular systolic pressure
PAT/PET: the ratio of pulmonary artery blood flow acceleration time to pulmonary artery blood ejection time
MDA: malondialdehyde
DMOG: dimethyloxallyl glycine
α-SMA: α-smooth muscle actin
